# Muscle-Preserving Versus Conventional Midline Expansive Open-Door Laminoplasty for Multilevel Cervical Spondylotic Myelopathy: A 10-Year Comparative Cohort Study

**DOI:** 10.3390/medicina62101902

**Published:** 2026-10-01

**Authors:** Hao-Wen Chen, Kuang-Ting Yeh, Wen-Tien Wu

**Affiliations:** 1 Department of Orthopedics, Hualien Tzu Chi Hospital, Buddhist Tzu Chi Medical Foundation, Hualien 970473, Taiwan; charliechen728@tzuchi.com.tw (H.-W.C.);; 2 School of Medicine, Tzu Chi University, Hualien 970374, Taiwan; 3 Institute of Medical Sciences, Tzu Chi University, Hualien 970374, Taiwan; 4 Graduate Institute of Clinical Pharmacy, Tzu Chi University, Hualien 970374, Taiwan; 5 Department of Medical Education, Hualien Tzu Chi Hospital, Buddhist Tzu Chi Medical Foundation, Hualien 970473, Taiwan

**Keywords:** laminoplasty, spondylosis, spinal cord compression, cervical vertebrae, treatment outcome, follow-up studies

## Abstract

*Background and Objectives*: Conventional midline expansive open-door laminoplasty (EODL) may disrupt the posterior cervical extensor mechanism. This study compared 10-year outcomes after conventional midline and unilateral para-midline muscle-preserving EODL. *Materials and Methods*: This retrospective cohort included 100 adults who underwent EODL for multilevel cervical spondylotic myelopathy from 1 January 2011 through 31 December 2015 (42 conventional and 58 muscle-preserving). At 10 years, 94 patients completed clinical assessments and 53 underwent MRI examinations. The primary outcome was the postoperative trajectory of the 17-point Japanese Orthopaedic Association (JOA) score from 3–6 months through 10 years. Models adjusted for age, sex, baseline outcome, and decompressed levels; sensitivity models additionally adjusted for operative year. *Results*: After operative-year adjustment, group-by-time interactions were significant for JOA (F(4, 385.749) = 9.094, *p* < 0.001), visual analog scale (VAS; F(4, 384.457) = 5.559, *p* < 0.001), and Neck Disability Index (NDI; F(4, 396.021) = 5.109, *p* = 0.001). At 10 years, adjusted means in the muscle-preserving versus conventional groups were 10.121 vs. 8.738 for JOA (difference, 1.383; 95% CI, 0.867–1.899), 3.718 vs. 5.946 for VAS (difference, −2.228; 95% CI, −2.643 to −1.813), and 15.395 vs. 20.592 for NDI (difference, −5.197; 95% CI, −6.952 to −3.442; all *p* < 0.001). Observed 10-year lordosis was 4.73° vs. −3.04°, sagittal vertical axis was 18.83 vs. 38.22 mm, and range of motion was 14.55° vs. 8.57° (all *p* < 0.001). Adjusted normalized paraspinal cross-sectional area was 7.840 vs. 5.591 (difference, 2.250; 95% CI, 1.752–2.748; *p* < 0.001). Operative time, blood loss, and hospital stay were lower with the muscle-preserving approach (88.89 vs. 121.82 min, 169.48 vs. 233.33 mL, and 6.12 vs. 7.19 days; all *p* < 0.001). Reoperation occurred in 4/58 vs. 6/42 patients (*p* = 0.314); the study was not powered to establish safety equivalence. *Conclusions*: The muscle-preserving approach was associated with more favorable intermediate- and long-term clinical, radiographic, and muscle-morphology outcomes. These associations persisted after operative-year adjustment for the clinical and CSA outcomes but do not establish causality because residual temporal and treatment-selection confounding remain possible.

## 1. Introduction

Degenerative cervical myelopathy (DCM) is a progressive spinal cord disorder caused by age-related narrowing of the cervical canal. Minimum North American estimates place its incidence and prevalence at approximately 4.1 and 60.5 per 100,000 persons, respectively, whereas United Kingdom modeling estimated a mean diagnosed prevalence of 0.19%, suggesting substantial under-recognition, particularly among older adults [1,2]. Common manifestations include loss of hand dexterity, upper-extremity numbness or paresthesia, gait imbalance, lower-extremity stiffness or weakness, falls, and, in advanced disease, sphincter dysfunction. Progressive neurological impairment can restrict mobility, self-care, employment, and independence; delayed diagnosis may permit irreversible disability. When neurological deterioration or function-limiting myelopathy is present, decompressive surgery is generally considered to prevent further spinal cord injury. Among posterior strategies, EODL provides multilevel decompression while retaining motion and posterior bony elements [3]. Originally developed by Hirabayashi, the procedure creates an opening-side trough and a contralateral hinge, permitting laminar elevation and posterior cord drift without routine fusion [4]. It is most often used for compression across three or more levels in patients without fixed kyphosis, major instability, or dominant focal anterior pathology [3,4]. Conventional midline exposure, however, may contribute to axial pain, loss of lordosis, reduced cervical range of motion (ROM), and paraspinal muscle atrophy [5,6,7]. Disruption of the semispinalis cervicis and other components of the posterior tension band may compromise dynamic stability and sagittal alignment [7,8]. Muscle-preserving modifications therefore use unilateral exposure, limited detachment, or alternative corridors to reduce injury to the deep extensors [9,10,11,12,13,14,15,16]. Although short- and mid-term findings are encouraging, evidence for durable neurological, radiographic, and muscle-morphology benefits remains limited and potentially susceptible to selection and operative-era confounding.

Accordingly, this study compared 10-year clinical, radiographic, and paraspinal muscle outcomes after unilateral para-midline muscle-preserving versus conventional midline EODL for multilevel cervical spondylotic myelopathy. The principal objective was to determine whether postoperative JOA trajectories differed between the techniques after accounting for baseline status and prespecified covariates; secondary objectives evaluated pain, disability, sagittal alignment, ROM, normalized paraspinal cross-sectional area (CSA), perioperative outcomes, and adverse events.

## 2. Materials and Methods

### 2.1. Study Design, Setting, and Ethics

This single-center retrospective comparative cohort study was approved by the Ethics Committee of Hualien Tzu Chi Hospital on 2 May 2023 (IRB112-054-B). The requirement for individual informed consent was waived because the study used existing clinical and imaging records. The institutional surgical registry covered procedures performed from 1 January 2011 through 31 December 2021. To ensure an equal opportunity to reach the 10-year assessment, eligibility was restricted to index operations performed from 1 January 2011 through 31 December 2015; follow-up was censored at each patient’s 10-year postoperative assessment.

### 2.2. Participants

Of 552 adults who underwent EODL during the registry period, 100 met the eligibility criteria. Inclusion required (1) clinical myelopathy with preoperative MRI-confirmed cervical stenosis involving at least three levels between C2 and C7; (2) a nonfixed cervical sagittal profile, defined as neutral or lordotic alignment or correction of any local kyphosis to at least 0° on dynamic extension radiography (local kyphosis that remained <0° on extension was classified as fixed kyphosis); and (3) complete baseline data, at least one postoperative assessment, and sufficient elapsed time to reach the 10-year assessment. All included patients underwent preoperative MRI; CT was not required for eligibility. C2–C7 Cobb angles were measured in all participants and are reported at baseline and longitudinally. Exclusion criteria were prior cervical decompression surgery, infectious, traumatic, or neoplastic pathology, rigid kyphotic deformity requiring osteotomy, a combined anterior–posterior index procedure, or surgery after 31 December 2015.

### 2.3. Surgical Techniques and Perioperative Care

Eligible patients were treated using one of two standardized surgical techniques: conventional midline EODL (Con group, *n* = 42) or unilateral para-midline muscle-preserving EODL (MP group, *n* = 58). The technique of surgical approach was selected according to surgeon preference, patient-specific considerations, and prevailing practice patterns rather than by random allocation. Both techniques were used during every calendar year from 2011 through 2015, and there was no discrete temporal cutoff separating the two procedures. Nevertheless, because treatment allocation was nonrandomized, temporal and indication-related confounding could not be excluded. In both groups, the decompression levels were selected according to the craniocaudal extent of spinal cord compression on preoperative imaging. The same open-door laminoplasty principles were applied at each treated level. The opening and hinge gutters were created approximately 15–20 mm lateral to the midline, with adjustment for individual anatomy at the relatively thin portion of the lamina while remaining medial to the facet joint. On the opening side, a bicortical gutter was created using a 3-mm high-speed cutting burr. On the contralateral hinge side, a 2-mm high-speed cutting burr was used to remove the dorsal cortex while preserving the ventral cortex. The lamina was then elevated posteriorly, producing a controlled greenstick fracture of the remaining ventral cortex at the hinge.

Canal expansion was standardized using a commercially manufactured five-hole miniplate, which was contoured to bridge the opening gutter and fixed with miniscrews. The intended posterior displacement of the elevated lamina was approximately 8–10 mm, corresponding to the standardized span between the plate fixation points. The same intended expansion range and fixation principles were applied in both groups. Adequacy of decompression was assessed intraoperatively according to laminar elevation, restoration of the posterior canal space, and the absence of visible residual constriction across the intended decompression levels.

C3 was treated with laminoplasty rather than laminectomy in all patients to preserve the posterior osseous structure and provide a potential attachment site for the posterior musculature. The ligamentum flavum at the C2–C3 junction was removed to minimize junctional constriction. In patients with spinal cord tenting or residual central stenosis at this junction on preoperative T2-weighted MRI, additional dome-shaped undercutting of the ventral aspect of the inferior C2 lamina was performed.

In the conventional technique, a standard posterior midline approach was used, with subperiosteal detachment of the bilateral paraspinal musculature to expose the indicated posterior elements. Detachment of the multifidus and semispinalis cervicis muscles, particularly at C2, was repaired when technically feasible. In contrast, the MP technique used a unilateral para-midline incision and splitting of the lateral raphe to develop a surgical corridor between the posterior cervical fascia and the medial paraspinal muscle belly. This approach preserved the contralateral musculature and minimized detachment of the posterior extensor insertions, particularly the semispinalis cervicis insertion at C2. Thus, the principal intended difference between the procedures was the route and extent of posterior muscular dissection rather than the planned laminoplasty levels or magnitude of laminar elevation.

All patients received standardized perioperative management, including multimodal analgesia, short-term cervical collar use for comfort, and early mobilization. Postoperative rehabilitation emphasized scapulothoracic coordination and progressive strengthening of the deep cervical extensor muscles.

### 2.4. Outcomes and Follow-Up

Clinical outcomes were assessed at baseline, 3–6 months, and 1, 2, 5, and 10 years postoperatively. The primary outcome was the longitudinal postoperative trajectory of the original 17-point Japanese Orthopaedic Association (JOA) score from 3–6 months through 10 years [17]. The group-by-time interaction was the principal parameter of interest; the adjusted 10-year between-group contrast was a time-specific model-derived comparison rather than a separate change-score endpoint. Secondary clinical outcomes were neck-pain VAS (0–10) and NDI (0–100) [18,19]. Secondary radiographic outcomes were C2–C7 Cobb lordosis, C2–C7 sagittal vertical axis (SVA), and C2–C7 ROM. Perioperative outcomes and adverse events, including C5 palsy, wound complications, implant-related events, and reoperations, were recorded through final follow-up.

Before the final reanalysis, all available JOA entries and cervical ROM measurements were reverified against the original medical records, clinical assessment documents, and radiographic records. Discrepancies arising during assembly of the analytical database were corrected before the verified dataset was locked. JOA values were required to fall within the valid 0–17 range. Missing observations were retained as missing and were not imputed. Descriptive summaries, longitudinal models, responder analyses, tables, figures, and manuscript text affected by the corrected variables were regenerated using the verified dataset.

### 2.5. Imaging and Measurement Procedures

Paraspinal muscle morphology was evaluated on axial T2-weighted magnetic resonance images at the mid-C5 vertebral level, or at the nearest technically suitable axial slice. The bilateral multifidus and semispinalis cervicis muscles were manually delineated using region-of-interest planimetry, and their combined cross-sectional area was recorded as the C5 paraspinal muscle CSA. The cross-sectional area of the corresponding C5 vertebral body was measured on the same axial image. Normalized CSA was calculated for each examination as the paraspinal muscle CSA divided by the corresponding vertebral-body area, thereby reducing variability related to patient size and image scaling. Paraspinal muscle CSA and vertebral-body area were reported in square millimeters, whereas normalized CSA was expressed as a dimensionless ratio.

Standing neutral, flexion, and extension lateral radiographs were obtained with horizontal gaze. C2–C7 lordosis was measured as the Cobb angle between the inferior endplates of C2 and C7; SVA was measured as the horizontal distance from the C2 centroid plumb line to the posterosuperior corner of C7; and ROM was calculated as the difference between extension and flexion C2–C7 angles [20]. MRI examinations were acquired on 1.5-T or 3-T systems with axial slice thickness ≤4 mm. Imaging measurements were performed independently by two observers blinded to surgical group.

### 2.6. Statistical Analysis

Continuous variables are reported as mean ± SD (normally distributed) or median with IQR (non-normally distributed), based on the Shapiro–Wilk test; categorical variables are reported as counts and percentages. Baseline characteristics were compared between surgical groups using independent-samples *t*-tests or Mann–Whitney U tests for continuous variables and Pearson’s chi-squared or Fisher’s exact tests for categorical variables. Operative year, being ordinal and non-normally distributed, was summarized by annual counts/percentages and median (IQR) per group and compared using the Mann–Whitney U test. Longitudinal clinical and radiographic outcomes were modeled using linear mixed-effects models with surgical group, time, and their interaction as fixed effects; the group-by-time interaction was the primary parameter of interest. Models were adjusted a priori for age, sex, baseline outcome value, and number of decompressed levels.

Sensitivity models additionally adjusted for operative year (continuous, coded as years since 2011). For JOA, the random-intercept model failed to converge (boundary variance estimate, non-positive-definite Hessian); it was refitted as a repeated-measures model with compound-symmetry covariance (REML). The VAS model retained a random intercept (REML). The NDI random-intercept model showed the same boundary problem and was similarly refitted with compound-symmetry covariance (ML). Denominator degrees of freedom used the Satterthwaite approximation. Adjusted marginal means and 95% CIs were obtained per group and time point, with MP-versus-conventional contrasts (Sidak-adjusted).

Clinical relevance was assessed via MCID achievement: ≥3-point JOA improvement (conservatively derived from Kato et al.’s 2.5-point threshold [21], given whole-point recording), ≥2-point VAS reduction, and ≥7-point NDI reduction. Responder proportions were compared using Fisher’s exact test and treated as exploratory given multiple comparisons. Hirabayashi JOA recovery rate—[(postoperative − baseline JOA)/(17 − baseline JOA)] × 100%—was calculated per patient and assessed as a supplementary measure for comparison with prior laminoplasty studies (no patient had baseline JOA = 17). For CSA analysis, observed C5 paraspinal CSA, vertebral-body area, and normalized CSA were summarized as mean ± SD at baseline and 3, 5, and 10 years, with available MRI counts per time point. Normalized CSA was analyzed using a repeated-measures mixed model (group, time, interaction; compound-symmetry covariance), adjusted for age, sex, baseline normalized CSA, decompressed levels, and operative year (ML). Marginal means, 95% CIs, and between-group contrasts were derived at each time point.

All models included available observations under a missing-at-random assumption without imputation; this assumption could not be verified and was considered when interpreting late-follow-up results. Model assumptions were checked via residual and residual-versus-fitted plots. Complications and reoperations were compared using Fisher’s exact test given small event counts; these analyses were descriptive/exploratory, with no prespecified equivalence margin. No a priori sample-size calculation was performed, as all eligible consecutive patients were included. A post hoc sensitivity calculation indicated the 10-year complete-case sample (54 MP, 40 conventional) provided 80% power (two-sided α = 0.05) to detect a standardized difference of ~0.59.

Analyses were performed using R 4.3.0 (R Foundation, Vienna, Austria) and SPSS 27.0 (IBM Corp., Armonk, NY, USA). Two-sided *p* < 0.05 was considered significant.

## 3. Results

### 3.1. Cohort Characteristics, Follow-Up, and Perioperative Outcomes

**Figure 1:**
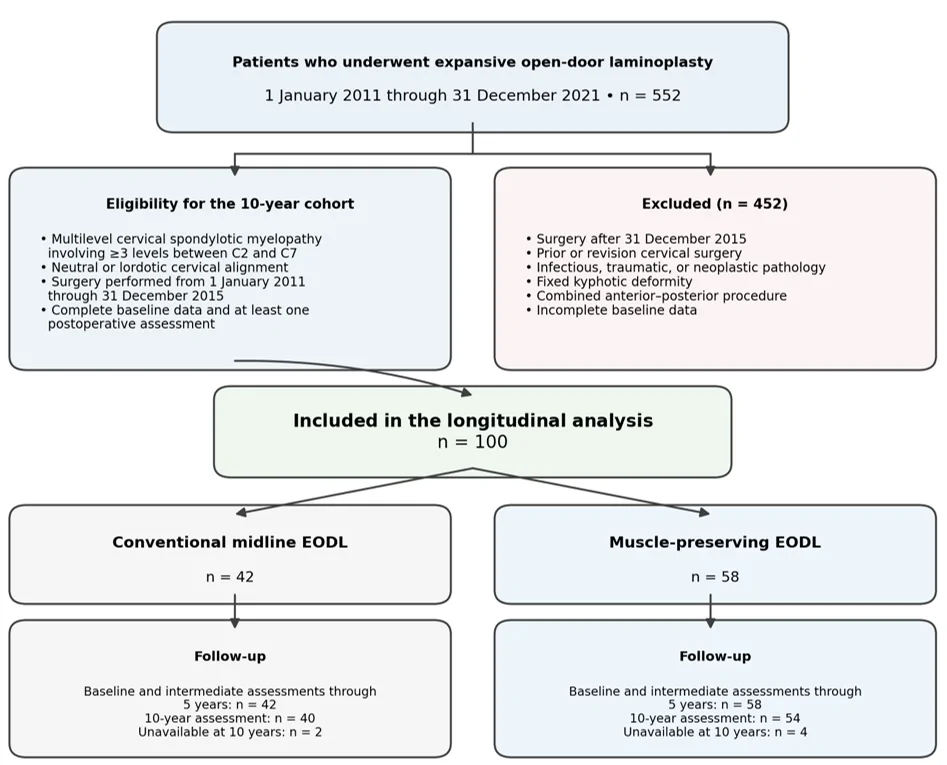
Flow diagram of cohort selection and follow-up.

From 1 January 2011 through 31 December 2021, 552 patients underwent EODL at the institution. Eligibility screening was restricted to index procedures performed from 1 January 2011 through 31 December 2015 (Figure 1). After application of the predefined criteria, 100 patients were included in the longitudinal analysis (58 MP and 42 conventional). All 100 contributed clinical observations through the 5-year assessment. At 10 years, six patients were unavailable (four MP and two conventional), leaving 94 patients with clinical data (54 MP and 40 conventional).

Baseline demographic and clinical characteristics, including age, sex, symptom duration, number of decompressed levels, preoperative JOA, VAS, and NDI scores, cervical alignment, and ROM, did not show statistically detectable between-group differences (Table 1). Both techniques were used in every calendar year from 2011 through 2015. In the conventional group, 12 (28.6%), 9 (21.4%), 5 (11.9%), 10 (23.8%), and 6 (14.3%) patients underwent surgery in 2011, 2012, 2013, 2014, and 2015, respectively; the corresponding MP counts were 6 (10.3%), 11 (19.0%), 15 (25.9%), 16 (27.6%), and 10 (17.2%). Median operative year was 2012.5 (IQR, 2011–2014) in the conventional group and 2013 (IQR, 2012–2014) in the MP group (Mann–Whitney U = 980.0, Z = −1.699, *p* = 0.089; Table 1 and Supplementary Table S1). This temporal overlap does not exclude residual era-related confounding.

Perioperative metrics differed between the techniques. The MP group had shorter operative time (88.89 ± 24.23 vs. 121.82 ± 36.24 min), lower estimated blood loss (169.48 ± 68.27 vs. 233.33 ± 61.27 mL), and shorter hospital stay (6.12 ± 1.33 vs. 7.19 ± 1.57 days; all *p* < 0.001; Table 2).

### 3.2. Clinical Outcomes

Both groups demonstrated early postoperative improvement in JOA, VAS, and NDI scores. However, the magnitude and timing of the between-group differences varied across outcomes.

In the operative-year-adjusted repeated-measures mixed model, the group-by-time interaction was significant for JOA score (F(4, 385.749) = 9.094, *p* < 0.001), indicating different postoperative trajectories after adjustment for age, sex, baseline JOA, number of decompressed levels, and operative year. Operative year was not significantly associated with postoperative JOA (β = −0.025 points per year; 95% CI, −0.110 to 0.060; *p* = 0.567).

The adjusted JOA scores did not differ significantly between groups at 3–6 months (MP minus conventional, 0.217 points; 95% CI: −0.284 to 0.718, *p* = 0.395) or 1 year (−0.005 points; 95% CI: −0.505 to 0.496, *p* = 0.985). Differences favoring the MP group emerged at 2 years (1.643 points; 95% CI: 1.142–2.144, *p* < 0.001) and persisted at 5 years (1.435 points; 95% CI: 0.935–1.936, *p* < 0.001). At 10 years, the adjusted mean JOA score was 10.121 (95% CI: 9.786–10.456) in the MP group and 8.738 (95% CI: 8.347–9.128) in the conventional group, corresponding to an adjusted difference of 1.383 points (95% CI: 0.867–1.899, *p* < 0.001). Thus, the adjusted JOA results did not demonstrate a uniform early advantage but showed divergence between the groups from the 2-year assessment onward.

The group-by-time interaction also remained significant after operative-year adjustment for VAS (F(4, 384.457) = 5.559, *p* < 0.001) and NDI (F(4, 396.021) = 5.109, *p* = 0.001). Later operative year was associated with slightly higher postoperative VAS scores (β = 0.134 points per year; 95% CI, 0.056–0.213; *p* = 0.001) and NDI scores (β = 0.266 points per year; 95% CI, 0.002–0.531; *p* = 0.049). At 10 years, adjusted mean VAS scores were 3.718 (95% CI, 3.448–3.987) in the MP group and 5.946 (95% CI, 5.632–6.260) in the conventional group (adjusted difference, −2.228; 95% CI, −2.643 to −1.813; *p* < 0.001). The corresponding adjusted NDI means were 15.395 and 20.592, respectively (adjusted difference, −5.197; 95% CI, −6.952 to −3.442; *p* < 0.001). Although statistically significant, the adjusted 10-year NDI difference did not reach the prespecified 7-point threshold for clinical importance. Complete model results, operative-year coefficients, estimated marginal means, and time-specific contrasts are presented in Table S2.

**Figure 2:**
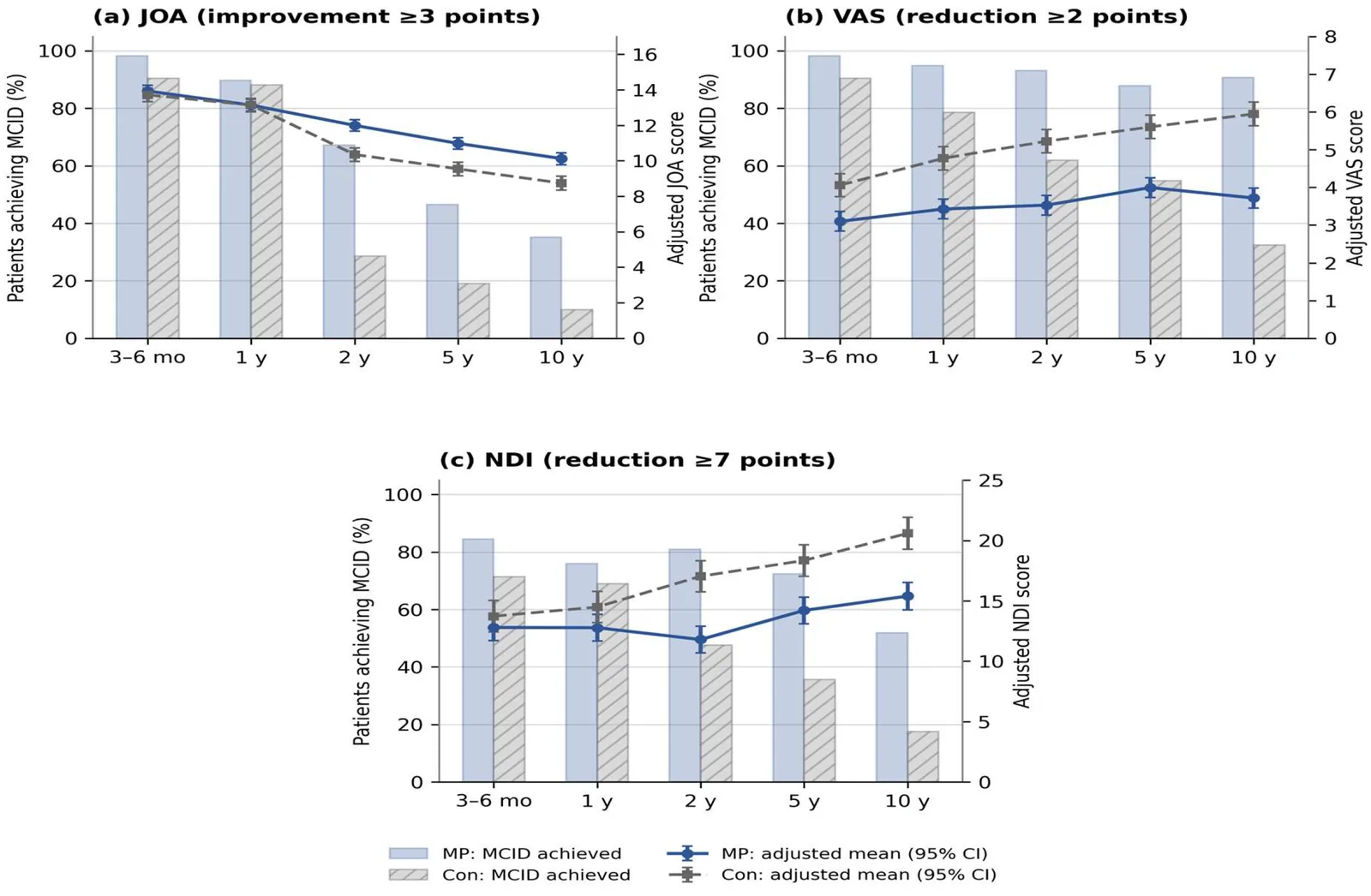
Longitudinal clinical outcomes and MCID achievement. Bars show the patient-level proportions achieving MCID at each postoperative assessment; lines show operative-year-adjusted estimated marginal means with 95% confidence intervals. MCID thresholds were a ≥3-point improvement for JOA, a ≥2-point reduction for VAS, and a ≥7-point reduction for NDI. Adjusted models included age, sex, baseline score, number of decompressed levels, and operative year. Con, conventional midline; JOA, Japanese Orthopaedic Association; MCID, minimal clinically important difference; MP, muscle-preserving; NDI, Neck Disability Index; VAS, visual analog scale.

Patient-level responder analyses provided complementary information regarding clinical relevance (Table 3 and Figure 2). Using an improvement of ≥3 whole JOA points as the operational threshold corresponding to the published 2.5-point MCID, the proportions achieving the JOA MCID did not differ significantly between the MP and conventional groups at 3–6 months (98.3% vs. 90.5%, *p* = 0.158) or 1 year (89.7% vs. 88.1%, *p* = 1.000). The proportions were higher in the MP group at 2 years (67.2% vs. 28.6%, *p* < 0.001), 5 years (46.6% vs. 19.0%, *p* = 0.006), and 10 years (35.2% vs. 10.0%, *p* = 0.007).

The early responder patterns, however, were not uniform across the three clinical outcomes. At 3–6 months, MCID achievement did not differ significantly between groups for JOA, VAS (98.3% vs. 90.5%, *p* = 0.158), or NDI (84.5% vs. 71.4%, *p* = 0.139). At 1 year, no statistically significant between-group difference was detected for JOA or NDI (75.9% vs. 69.0%, *p* = 0.498), whereas the VAS responder rate was higher in the MP group (94.8% vs. 78.6%, *p* = 0.026). From 2 years onward, MCID achievement was consistently higher in the MP group across JOA, VAS, and NDI. Thus, these responder analyses do not indicate formal early equivalence or a uniform early MP advantage; rather, they suggest that the differences favoring the MP group became more consistent during intermediate and long-term follow-up. Because these patient-level MCID analyses were supplementary and exploratory, the time-specific *p* values should be interpreted cautiously in view of the multiple comparisons.

The Hirabayashi JOA recovery rate was calculated as a supplementary measure accounting for baseline severity and the 17-point ceiling (Table S3). Mean recovery rates in the MP and conventional groups were 62.3 ± 15.8% and 60.1 ± 17.3% at 3–6 months, 53.0 ± 16.9% and 53.2 ± 17.2% at 1 year, 38.9 ± 16.7% and 20.3 ± 15.8% at 2 years, 26.2 ± 16.6% and 10.4 ± 15.8% at 5 years, and 15.8 ± 21.3% and −0.7 ± 26.6% at 10 years, respectively. At 10 years, the unadjusted between-group difference was 16.5 percentage points (95% CI, 6.3–26.7; *p* = 0.002). These supplementary results were consistent with the longitudinal model in showing comparable early neurological recovery followed by greater maintenance of improvement in the MP group.

Overall, the clinical results indicate that the principal between-group differences involved the longer-term trajectories rather than a uniform advantage immediately after surgery. These associations persisted after adjustment for operative year and the other prespecified covariates, although residual confounding arising from the retrospective, nonrandomized design cannot be excluded.

### 3.3. Radiographic Outcomes

**Figure 3:**
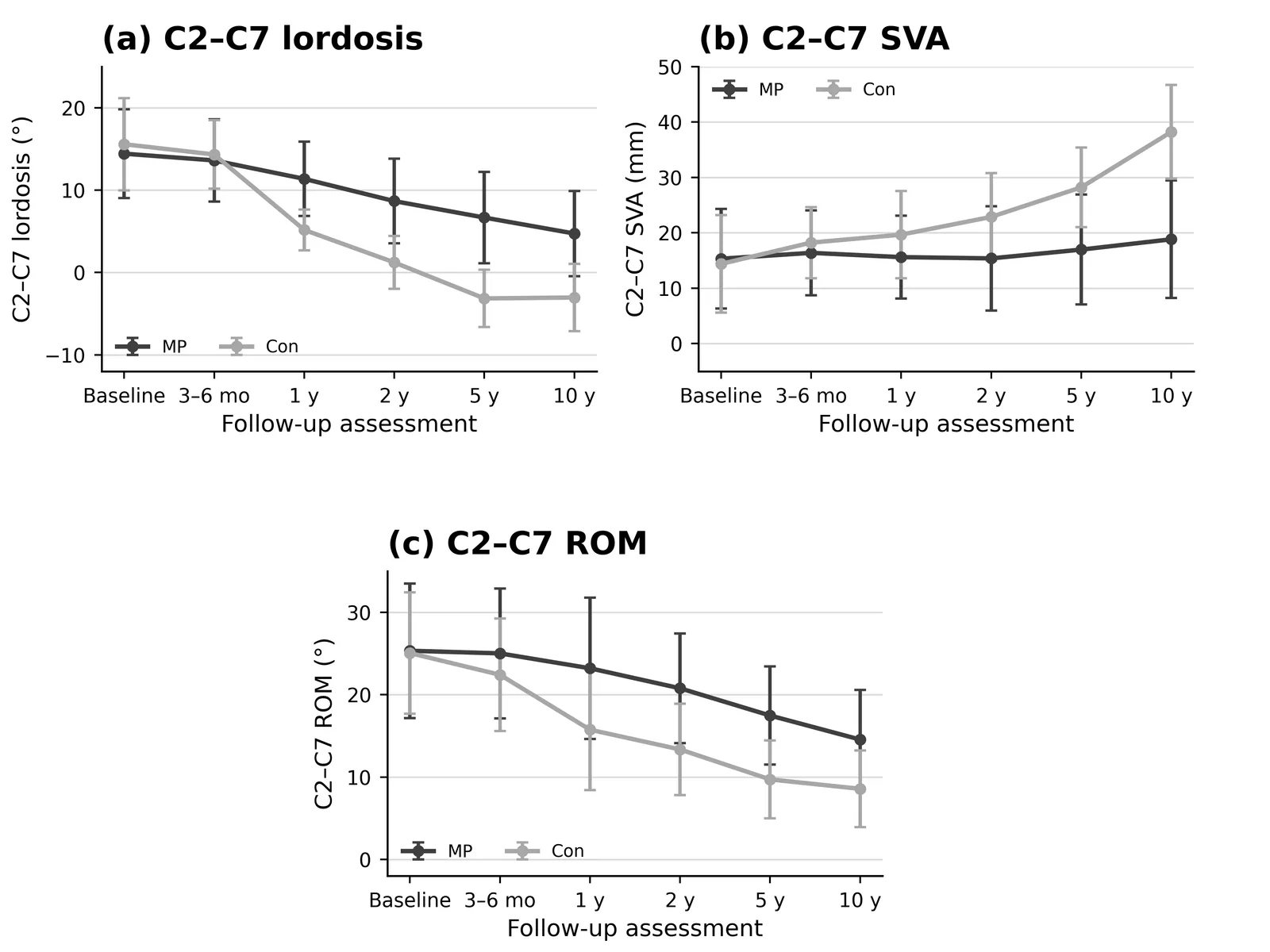
Observed C2–C7 lordosis, sagittal vertical axis (SVA), and range of motion (ROM) by surgical group and follow-up assessment. Points and error bars show means ± SD. Con, conventional midline; MP, muscle-preserving.

Observed radiographic trajectories diverged over follow-up (Table 4 and Figure 3). At 3–6 months, between-group differences in lordosis, SVA, and ROM were not statistically significant. From 1 year onward, the MP group showed greater observed lordosis and ROM and lower SVA than the conventional group (all time-specific *p* < 0.05). The group-by-time interactions were significant in the prespecified longitudinal models (all *p* < 0.001). These secondary findings are consistent with better long-term preservation of cervical alignment and mobility in the MP group but remain susceptible to residual confounding.

### 3.4. Paraspinal Muscle Morphology

Observed cohort-level C5 paraspinal muscle measurements and adjusted longitudinal normalized CSA estimates are presented in Table 5. At baseline, mean normalized CSA was 10.275 ± 1.285 in the conventional group and 9.880 ± 0.911 in the MP group. At 3 years, the corresponding values were 7.911 ± 0.870 and 8.837 ± 1.131; at 5 years, they were 6.613 ± 0.793 and 8.100 ± 1.104; and at 10 years, they were 5.630 ± 0.719 and 7.892 ± 0.715, respectively. Based on the observed group means, normalized CSA decreased from baseline by 23.0%, 35.6%, and 45.2% at 3, 5, and 10 years in the conventional group and by 10.6%, 18.0%, and 20.1%, respectively, in the MP group. The corresponding C5 paraspinal muscle CSA and vertebral-body area measurements are provided in Table 5.

In the operative-year-adjusted longitudinal model, the normalized CSA trajectory differed significantly between groups (global group-by-time Wald χ^2^(2) = 17.336, *p* < 0.001). At 3 years, the adjusted mean normalized CSA was 7.908 in the conventional group and 8.848 in the MP group, corresponding to an adjusted difference of 0.940 (95% CI, 0.565–1.315; *p* < 0.001). At 5 years, the adjusted means were 6.576 and 8.058, respectively, with an adjusted difference of 1.482 (95% CI, 1.028–1.936; *p* < 0.001). At 10 years, the adjusted means were 5.591 and 7.840, respectively, yielding an adjusted difference of 2.250 (95% CI, 1.752–2.748; *p* < 0.001). Operative year was not significantly associated with normalized CSA (β = −0.070 per year; 95% CI, −0.177 to 0.036; *p* = 0.197).

MRI examinations were available for all 100 patients at baseline and 3 years, 64 patients at 5 years, and 53 patients at 10 years. Specifically, the numbers of available examinations in the conventional and MP groups were 42 and 58 at baseline and 3 years, 33 and 31 at 5 years, and 27 and 26 at 10 years, respectively. Although the longitudinal model incorporated all available observations, the later CSA findings should be interpreted with consideration of possible selection related to follow-up MRI availability.

**Figure 4:**
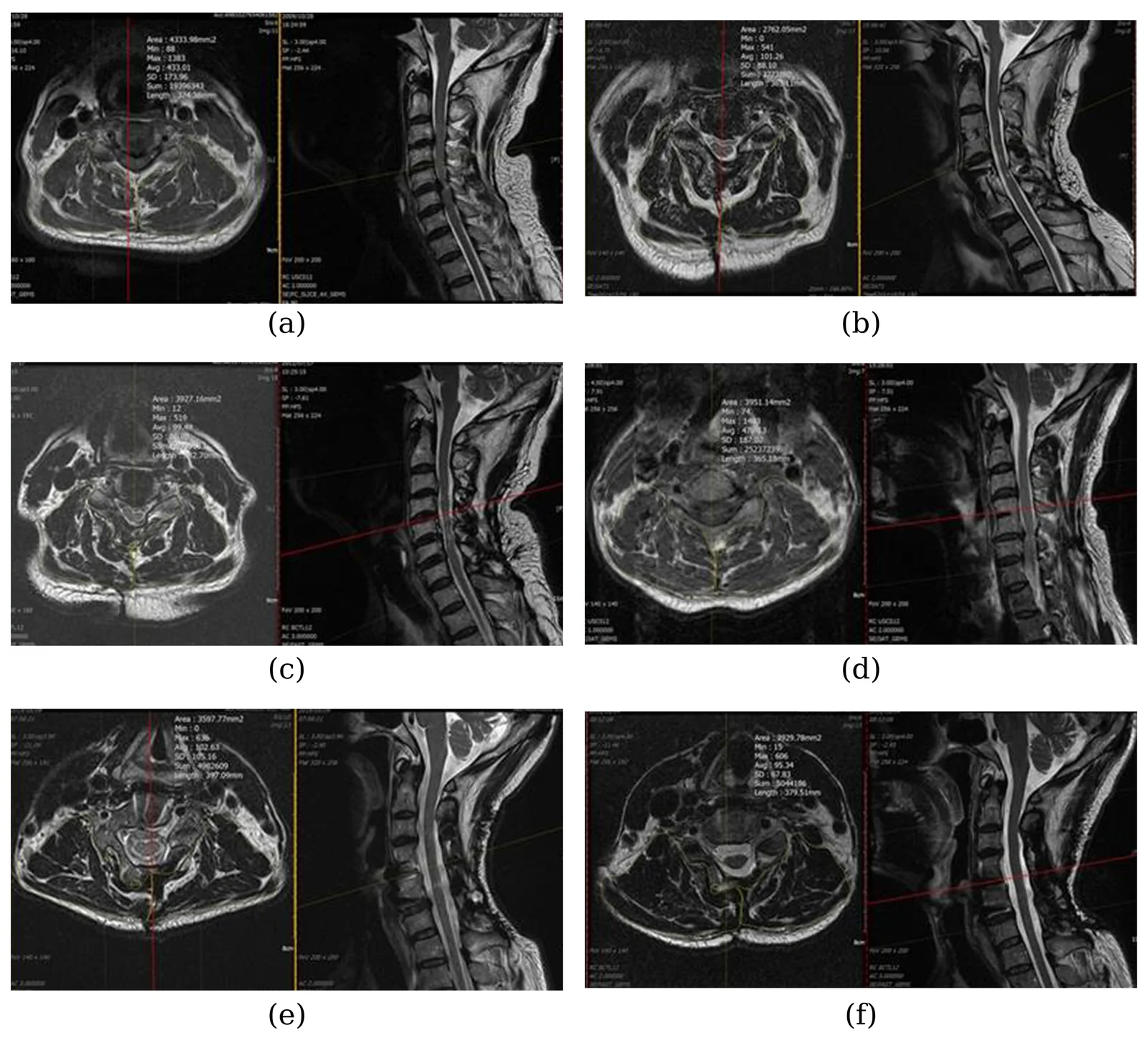
Illustrative examples of longitudinal C5 paraspinal muscle measurements. Axial T2-weighted magnetic resonance images illustrating region-of-interest measurement of the combined bilateral multifidus and semispinalis cervicis cross-sectional area at the C5 level and normalization to the corresponding vertebral-body area. The yellow/red frames identify the active image windows in the workstation, the red line marks the axial slice level on the sagittal localizer, and the overlaid symbols and numerical values are workstation-generated ROI measurements. Panels (**a**–**c**) show baseline, 3-year, and 10-year images from one patient in the conventional midline group, whereas panels (**d**–**f**) show the corresponding assessments from one patient in the muscle-preserving group. These individual cases were selected solely to illustrate the measurement method and longitudinal imaging appearance and should not be interpreted as representative of the magnitude of the cohort-level effect or as independent evidence of a treatment effect. Cohort-level descriptive statistics and longitudinal model estimates are presented in Table 5.

Figure 4 presents illustrative images from one patient in each surgical group to demonstrate the region-of-interest measurement method and longitudinal imaging appearance. These individual cases were not used to estimate the magnitude or statistical significance of the between-group differences.

### 3.5. Complications and Reoperations

Recorded adverse events and reoperations were infrequent in both groups. C5 palsy occurred in 1 of 58 patients (1.7%) in the MP group and 1 of 42 patients (2.4%) in the conventional group (Fisher’s exact *p* = 1.000). Wound complications occurred in 2 patients (3.4%) and 3 patients (7.1%), respectively (*p* = 0.647). Reoperation within 10 years was recorded in 4 patients (6.9%) in the MP group and 6 patients (14.3%) in the conventional group (*p* = 0.314). The indications for reoperation included adjacent-segment foraminal stenosis and spacer- or plate-related problems.

No statistically significant between-group differences were detected for these outcomes. Nevertheless, the small number of events resulted in limited statistical precision, and the analyses were descriptive and exploratory. Accordingly, these findings do not establish equivalence between the procedures and do not exclude potentially clinically meaningful differences in the risks of uncommon adverse events or reoperation.

## 4. Discussion

This study found that unilateral para-midline muscle-preserving EODL was associated with more favorable intermediate- and long-term clinical trajectories than conventional midline EODL. Both groups improved early, but the early pattern varied by outcome and analytic measure. Adjusted JOA scores and JOA responder rates did not differ significantly during the first postoperative year; adjusted VAS favored MP from the first assessment, whereas early VAS responder and NDI findings were less uniform. From 2 years onward, differences favoring MP became more consistent across neurological function, pain, and disability. These results should not be interpreted as formal early equivalence or as evidence of a uniform immediate advantage.

Several mechanisms may underlie the later divergence. Limited posterior dissection may reduce injury to the semispinalis cervicis and other deep extensors, which could help preserve the posterior tension band, sagittal alignment, and cervical mobility. Consistent with this hypothesis, the MP group maintained higher cohort-level normalized CSA and more favorable observed alignment and ROM at later assessments. The MP approach was also associated with shorter operative time, lower blood loss, and shorter hospital stay. However, the study did not formally test mediation, and differences in canal geometry, hinge behavior, patient selection, or unmeasured care processes may also have contributed. Figure 4 is illustrative only; inferences regarding muscle morphology are based on the cohort-level data and longitudinal model in Table 5.

The present findings are consistent with prior comparative and longitudinal studies demonstrating that EODL provides reliable neurological recovery and durable decompression in patients with multilevel cervical spondylotic myelopathy [14,15,22,23]. Consistent improvements in JOA scores across studies confirm the effectiveness of posterior decompression as a treatment strategy for this condition. Muscle-preserving laminoplasty techniques have shown favorable midterm outcomes, including high neurological recovery rates and effective canal expansion with posterior cord shift. Motion preservation remains a key advantage of laminoplasty compared with fusion-based procedures. Modified laminoplasty has been reported to preserve approximately 85% of cervical ROM, whereas hybrid strategies combining laminoplasty with anterior fusion retain less motion but may better address focal anterior pathology or segmental kyphosis [10,15]. These findings collectively support laminoplasty as a preferred motion-preserving strategy when sagittal alignment is acceptable and instability is absent [8,11]. Midterm and longer follow-up cohorts of modified laminoplasty further demonstrate sustained neurological improvement, preservation of cervical motion in a substantial proportion of patients, and low rates of door reclosure when modern fixation techniques are employed [10,14,24]. Compared with multilevel anterior or instrumented posterior fusion, laminoplasty preserves greater cervical mobility while achieving comparable neurological recovery, although technique-specific risks must be carefully balanced against sagittal alignment considerations and the presence of focal anterior pathology [14,22,25,26].

Complications and reoperations represent important considerations in surgical decision-making. In the present cohort, no statistically significant between-group differences were detected in the recorded adverse events or reoperations. However, the event counts were small: C5 palsy occurred in one patient in each group, whereas reoperation within 10 years occurred in 4 patients (6.9%) in the MP group and 6 patients (14.3%) in the conventional group. These findings indicate an absence of a statistically detectable difference rather than evidence of equivalent safety. The study was not designed or powered to establish equivalence or noninferiority for these uncommon outcomes, and clinically meaningful differences therefore cannot be excluded. Larger studies with complication-specific power calculations or prespecified noninferiority margins are required to determine whether the procedures differ in the risks of uncommon adverse events or reoperation.

C5 palsy is a recognized complication following posterior decompression, although its incidence may be reduced with muscle-preserving techniques and selective foraminotomy [27]. Axial neck pain and paraspinal muscle atrophy are strongly associated with disruption of posterior cervical musculature, particularly detachment of the semispinalis cervicis and muscle insertions at C2 and C7 [23,25]. Techniques that preserve these structures have been shown to reduce axial symptoms and improve postoperative ROM [6,11,25]. Door reclosure and implant-related complications are uncommon with modern plate fixation systems, which provide stable canal expansion and may outperform suture-based techniques in maintaining decompression [24]. Nevertheless, postoperative kyphotic change remains a concern, particularly in patients with unfavorable preoperative sagittal alignment or disruption of the posterior tension band [28,29,30]. In such cases, adjunctive fusion or hybrid approaches may represent more appropriate surgical strategies. Recent technical advances—including muscle-sparing paraspinal approaches, rigid laminoplasty plate systems, unilateral or minimally invasive corridors, and alternative expansion geometries such as French-door and two-door techniques—aim to further reduce soft-tissue morbidity and improve the reliability of canal expansion [16,24,31]. Endoscopic and biportal adaptations remain in the early stages of development and are technically demanding, with limited long-term outcome data currently available [16,24,31].

A key contribution is the longitudinal quantification of paraspinal muscle morphology alongside clinical and radiographic outcomes. In the conventional group, larger CSA declines coincided with worsening sagittal alignment and the emergence of JOA differences from 2 years onward, suggesting—without proving—a link between posterior muscle integrity, cervical biomechanics, and neurological function. A muscle-preserving approach may suit multilevel posterior decompression with favorable alignment, while fusion or hybrid strategies remain appropriate for ventral pathology, instability, or kyphosis; prospective comparisons are needed before either approach can be preferentially recommended [10,14,15,22,24,32].

Because the MP procedure became more frequent in later years, sensitivity analyses adjusted the clinical and CSA models for operative year. Group-by-time interactions remained significant for JOA (F(4, 385.749) = 9.094, *p* < 0.001), VAS (F(4, 384.457) = 5.559, *p* < 0.001), NDI (F(4, 396.021) = 5.109, *p* = 0.001), and normalized CSA (Wald χ^2^(2) = 17.336, *p* < 0.001). Operative year itself was unrelated to JOA or CSA and only weakly associated with higher VAS/NDI scores, indicating that later surgery did not simply produce better outcomes—though calendar year imperfectly captures evolving perioperative care, rehabilitation, imaging, and surgeon experience, so residual confounding remains possible.

Strengths include up to 10 years of follow-up, comprehensive clinical and radiographic assessment, longitudinal MRI-based CSA quantification, and consistent surgical technique within a single institution.

Several limitations should be noted. First, the retrospective, nonrandomized design permits treatment-selection bias, as technique choice reflected surgeon preference and evolving practice rather than randomization; although models were adjusted for operative year, residual confounding from indication, surgeon experience, and unmeasured temporal factors cannot be excluded, so a causal effect of technique alone is not established. Second, data were transcribed from clinical and radiographic records; a source audit identified a systematic mapping error in the JOA dataset and one transcription error in the 10-year ROM data. All JOA and ROM entries were reverified, corrected, and reanalyzed, though residual transcription risk remains. Third, although decompression levels, technique, fixation, and laminar elevation (~8–10 mm) were standardized, canal-expansion symmetry and hinge healing were not systematically imaged, so unmeasured technical variation—beyond muscle preservation itself—may have contributed to the observed outcomes. Fourth, progressive attrition may limit representativeness: while 94 of 100 patients completed 10-year clinical follow-up, serial MRI was available for only 64 patients at 5 years and 53 at 10 years, with differential availability between groups, and the missing-at-random assumption cannot be verified. Fifth, variability in MRI timing, field strength, and slice positioning may affect measurement precision; vertebral-body normalization reduces but does not eliminate this, and CSA was assessed at a single axial level rather than volumetrically or for fatty infiltration—Figure 4 illustrates the measurement method only and does not represent cohort-level effects. Sixth, no a priori sample-size calculation was performed because all eligible consecutive patients were included; consequently, the sample may have been insufficient to detect smaller between-group differences, and the post hoc sensitivity calculation does not replace prospective sample-size planning. Finally, the study was underpowered for rare adverse events, and nonsignificant complication/reoperation findings reflect small event counts rather than demonstrated safety equivalence.

Future prospective studies should incorporate randomized or otherwise rigorously controlled treatment allocation, standardized rehabilitation and imaging protocols, prespecified serial MRI follow-up, and strategies to minimize missing imaging data. Advanced imaging approaches, including volumetric muscle assessment and fat-fraction mapping, together with electromyographic and patient-reported outcomes, may further clarify the functional significance of posterior extensor preservation and its association with long-term cervical biomechanics.

## 5. Conclusions

In this single-center retrospective cohort, unilateral para-midline muscle-preserving EODL was associated with more favorable intermediate- and long-term clinical trajectories and better preservation of cervical alignment, ROM, and paraspinal muscle CSA than conventional midline EODL. Adjusted JOA outcomes did not differ significantly during the first postoperative year, whereas differences favoring MP emerged from 2 years onward; the clinical and CSA associations persisted after operative-year adjustment. Because treatment allocation was nonrandom and residual temporal, selection, and technical confounding cannot be excluded, these findings do not establish causality and require confirmation in prospective comparative studies.

## Figures and Tables

**Table 1:** Baseline characteristics.

Variables	Muscle-Preserving (*n* = 58)	Conventional Midline (*n* = 42)	*p* Value
Age, years (mean ± SD)	64.48 ± 5.97	63.19 ± 5.39	0.261
Male, *n* (%)	30 (51.7%)	24 (57.1%)	0.685
Symptom duration, months	9.42 ± 4.29	9.31 ± 3.08	0.891
Levels decompressed, *n*	4.03 ± 0.59	4.10 ± 0.30	0.502
JOA, baseline	8.67 ± 1.07	8.52 ± 1.33	0.178
VAS-neck, baseline	7.17 ± 0.90	7.02 ± 1.09	0.473
NDI, baseline	23.33 ± 3.18	22.98 ± 3.69	0.620
C2–C7 lordosis, °	14.43 ± 5.38	15.58 ± 5.60	0.305
C2–C7 SVA, mm	15.32 ± 8.97	14.38 ± 8.80	0.603
C2–C7 ROM, °	25.34 ± 8.17	25.06 ± 7.38	0.856
Operative year, median (IQR)	2013 (2012–2014)	2012.5 (2011–2014)	0.089

Operative year was compared using the Mann–Whitney U test (U = 980.0, Z = −1.699). Both surgical techniques were represented in every calendar year of the study period. Abbreviations: SD, standard deviation; JOA, Japanese Orthopaedic Association; VAS, visual analog scale; NDI, neck disability index; SVA, sagittal vertical axis; ROM, range of motion.

**Table 2:** Perioperative metrics and complications.

Variables	Muscle-Preserving (*n* = 58)	Conventional Midline (*n* = 42)	*p* Value
Operative time, min	88.89 ± 24.23	121.82 ± 36.24	<0.001
Estimated blood loss, mL	169.48 ± 68.27	233.33 ± 61.27	<0.001
Length of stay, days	6.12 ± 1.33	7.19 ± 1.57	<0.001
C5 palsy, *n* (%)	1 (1.7%)	1 (2.4%)	1.000
Wound complication, *n* (%)	2 (3.4%)	3 (7.1%)	0.647
Reoperation within 10 y, *n* (%)	4 (6.9%)	6 (14.3%)	0.314

**Table 3:** Observed longitudinal clinical outcomes and proportions achieving MCID.

Time Point	Gr.	JOA	VAS	NDI	MCID JOA ≥ 3	MCID VAS ↓ ≥ 2	MCID NDI ↓ ≥ 7
Baseline	MP	8.67 ± 1.07	7.17 ± 0.90	23.33 ± 3.18	—	—	—
	Con	8.52 ± 1.33	7.02 ± 1.09	22.98 ± 3.69	—	—	—
3–6 months	MP	13.91 ± 1.23	3.14 ± 0.89	12.83 ± 3.03	57/58 (98.3%)	57/58 (98.3%)	49/58 (84.5%)
	Con	13.71 ± 1.33	4.00 ± 0.86	13.69 ± 3.25	38/42 (90.5%)	38/42 (90.5%)	30/42 (71.4%)
1 year	MP	13.12 ± 1.36	3.47 ± 0.96	12.81 ± 4.39	52/58 (89.7%)	55/58 (94.8%)	44/58 (75.9%)
	Con	13.14 ± 1.20	4.71 ± 0.86	14.45 ± 3.62	37/42 (88.1%)	33/42 (78.6%)	29/42 (69.0%)
2 years	MP	11.98 ± 1.25	3.57 ± 1.13	11.83 ± 3.84	39/58 (67.2%)	54/58 (93.1%)	47/58 (81.0%)
	Con	10.36 ± 1.19	5.17 ± 1.08	17.00 ± 4.43	12/42 (28.6%)	26/42 (61.9%)	20/42 (47.6%)
5 years	MP	10.97 ± 1.03	4.03 ± 1.11	14.24 ± 5.05	27/58 (46.6%)	51/58 (87.9%)	42/58 (72.4%)
	Con	9.55 ± 1.04	5.55 ± 1.15	18.31 ± 4.71	8/42 (19.0%)	23/42 (54.8%)	15/42 (35.7%)
10 years	MP	10.11 ± 1.45	3.72 ± 1.12	15.41 ± 5.75	19/54 (35.2%)	49/54 (90.7%)	28/54 (51.9%)
	Con	8.75 ± 1.32	5.88 ± 0.99	20.55 ± 4.41	4/40 (10.0%)	13/40 (32.5%)	7/40 (17.5%)

JOA MCID achievement was operationally defined as an improvement of ≥3 whole points because the published 2.5-point MCID could not be observed directly when JOA scores were recorded in whole-point increments. Values are mean ± SD. MCID thresholds used: JOA increase ≥ 3, VAS decrease ≥ 2, NDI decrease ≥ 7 from baseline.

**Table 4:** Radiographic parameters over time.

Time Point	Gr.	C2–C7 Lordosis (°)	*p* Value	C2–C7 SVA (mm)	*p* Value	C2–C7 ROM (°)	*p* Value
Baseline	MP	14.43 ± 5.38	0.305	15.32 ± 8.97	0.603	25.34 ± 8.17	0.856
	Con	15.58 ± 5.60		14.38 ± 8.80		25.06 ± 7.38	
3–6 months	MP	13.61 ± 4.99	0.436	16.38 ± 7.67	0.192	25.02 ± 7.89	0.081
	Con	14.33 ± 4.16		18.23 ± 6.41		22.42 ± 6.82	
1 year	MP	11.37 ± 4.52	<0.001	15.61 ± 7.49	0.011	23.21 ± 8.58	<0.001
	Con	5.17 ± 2.48		19.67 ± 7.89		15.75 ± 7.33	
2 years	MP	8.67 ± 5.14	<0.001	15.38 ± 9.43	<0.001	20.78 ± 6.66	<0.001
	Con	1.22 ± 3.21		22.87 ± 7.90		13.35 ± 5.55	
5 years	MP	6.67 ± 5.54	<0.001	16.97 ± 9.92	<0.001	17.47 ± 5.96	<0.001
	Con	−3.15 ± 3.48		28.21 ± 7.21		9.71 ± 4.73	
10 years	MP	4.73 ± 5.18	<0.001	18.83 ± 10.61	<0.001	14.55 ± 6.03	<0.001
	Con	−3.04 ± 4.07		38.22 ± 8.46		8.57 ± 4.65	

Values are observed means ± SD. The displayed *p* values are unadjusted time-specific between-group comparisons; longitudinal inference was based on the group-by-time interaction. Radiographic analyses were secondary and exploratory.

**Table 5:** Longitudinal C5 paraspinal muscle measurements and adjusted normalized CSA estimates.

(**A**) Observed C5 measurements by group and time point								
**Time Point**	**Con** **(** ***n*** **)**	**Paraspinal** **CSA (mm^2^)**	**C5 body** **Area (mm^2^)**	**Normalized CSA**	**MP** **(** ***n*** **)**	**Paraspinal** **CSA (mm^2^)**	**C5 body** **Area (mm^2^)**	**Normalized** **CSA**
		**Mean ± SD**				**Mean ± SD**		
Baseline	42	3815.4 ± 328.1	374.1 ± 30.4	10.275 ± 1.285	58	3749.7 ± 242.3	381.3 ± 27.7	9.880 ± 0.911
3 years	42	2992.1 ± 231.6	380.4 ± 29.5	7.911 ± 0.870	58	3359.6 ± 331.5	382.7 ± 30.3	8.837 ± 1.131
5 years	33	2498.8 ± 218.3	380.3 ± 29.7	6.613 ± 0.793	31	3119.8 ± 368.0	386.9 ± 25.3	8.100 ± 1.104
10 years	27	2154.3 ± 200.2	384.9 ± 26.9	5.630 ± 0.719	26	3010.3 ± 210.3	382.8 ± 26.0	7.892 ± 0.715
(**B**) Operative-year-adjusted longitudinal estimates of normalized CSA								
**Time Point**		**Conventional Adjusted Mean** **(95% CI)**		**MP Adjusted Mean** **(95% CI)**		**Adjusted Difference,** **MP − Conventional (95% CI)**		***p*** **Value**
3 years		7.908 (7.625–8.192)		8.848 (8.609–9.087)		0.940 (0.565–1.315)		<0.001
5 years		6.576 (6.245–6.907)		8.058 (7.723–8.393)		1.482 (1.028–1.936)		<0.001
10 years		5.591 (5.226–5.955)		7.840 (7.473–8.208)		2.250 (1.752–2.748)		<0.001
Global group-by-time interaction: Wald χ^2^(2) = 17.336, *p* < 0.001. Operative-year effect: β = −0.070 per year (95% CI, −0.177 to 0.036), *p* = 0.197.								
(**C**) Observed normalized CSA change from baseline								
**Group**		**3 Years**		**5 Years**		**10 Years**		
Conventional		−23.0%		−35.6%		−45.2%		
Muscle-preserving		−10.6%		−18.0%		−20.1%		

Data in (**A**) are observed means ± standard deviations, with the number of available MRI examinations reported separately for each group and time point. C5 paraspinal CSA represents the combined bilateral multifidus and semispinalis cervicis cross-sectional area. Normalized CSA was calculated as C5 paraspinal muscle CSA divided by the corresponding C5 vertebral-body area and is therefore dimensionless. (**B**,**C**) present model-based estimated marginal means and 95% confidence intervals adjusted for age, sex, baseline normalized CSA, number of decompressed levels, and operative year. Operative year was entered as a continuous variable coded as years since 2011. Between-group differences were calculated as muscle-preserving minus conventional. The repeated-measures model used a compound-symmetry covariance structure and maximum-likelihood estimation and incorporated all available postoperative observations under a missing-at-random assumption. CSA, cross-sectional area; CI, confidence interval; MP, muscle-preserving; SD, standard deviation.

## Data Availability

Deidentified data supporting the findings are available from the corresponding author upon reasonable request, subject to institutional and ethical requirements.

## Supplementary Materials

The following supporting information can be downloaded at https://www.mdpi.com/article/10.3390/medicina62101902/s1, Table S1: Distribution of operative year by surgical group; Table S2: Operative-year-adjusted longitudinal analyses of JOA, VAS, and NDI outcomes; Table S3: Hirabayashi JOA recovery rates by surgical group and postoperative assessment.

## Abbreviations

The following abbreviations are used in this manuscript:

Con	Conventional midline group
CSA	Cross-sectional area
EODL	Expansive open-door laminoplasty
IQR	Interquartile range
IRB	Institutional Review Board
JOA	Japanese Orthopaedic Association
MCID	Minimal clinically important difference
MCSM	Multilevel cervical spondylotic myelopathy
MP	Muscle-preserving group
MRI	Magnetic resonance imaging
NDI	Neck Disability Index
ROI	Region of interest
ROM	Range of motion
SD	Standard deviation
SVA	Sagittal vertical axis
VAS	Visual analog scale

## References

[1] Nouri A., Tetreault L., Singh A., Karadimas S.K., Fehlings M.G. (2015). Degenerative Cervical Myelopathy: Epidemiology, Genetics, and Pathogenesis. Spine.

[2] Grodzinski B., Stubbs D.J., Davies B.M. (2023). Most Degenerative Cervical Myelopathy Remains Undiagnosed, Particularly amongst the Elderly: Modelling the Prevalence of Degenerative Cervical Myelopathy in the United Kingdom. J. Neurol..

[3] Cho N., Mehta A.I., Vedantam A., Goodwin C.R., Agochukwu U.F., Grassner L., Nouri A., Aarabi B., Wilson J.R., Evaniew N. (2026). Techniques for Cervical Laminoplasty. Glob. Spine J..

[4] Hirabayashi K., Watanabe K., Wakano K., Suzuki N., Satomi K., Ishii Y. (1983). Expansive open-door laminoplasty for cervical spinal stenotic myelopathy. Spine.

[5] Gao Y., Jiang L. (2026). Clinical efficacy of posterior cervical c3 laminectomy combined with c7 underlying decompression and laminoplasty with support titanium plate fixation for cervical spondylotic myelopathy. BMC Musculoskelet. Disord..

[6] Qu R., Wang B., Pan S., Liu Z., Yang Y., Zhou H., Dang L., Sun Y., Zhou F., Jiang L. (2025). Preservation of short-term cervical posterior muscle volume and cervical alignment: A comparison between three types of modified muscle-sparing laminoplasty and conventional open-door laminoplasty for multilevel degenerative cervical myelopathy. BMC Musculoskelet. Disord..

[7] Lin C.R., Tsai S.H.L., Tsai P.A., Chen Y.J., Chen M.H., Tsai S.A., Hsu L.S., Lee K.H., Lee Z.Y., Kao F.C. (2025). What is the best strategy for C3 in open-door laminoplasty: Laminectomy versus laminoplasty—A systematic review and meta-analysis. Spine J..

[8] Kim J.H., Yuh W.T., Han J., Kim T., Lee C.H., Kim C.H., Choi Y., Chung C.K. (2023). Impact of C3 laminectomy on cervical sagittal alignment in cervical laminoplasty: A prospective, randomized controlled trial comparing clinical and radiological outcomes between C3 laminectomy with C4-C6 laminoplasty and C3-C6 laminoplasty. Spine J..

[9] Zhong H., Xu C., Wang H., Wang R., Wei L., Wu H., Wang X., Liu Y., Chen H., Yuan W. (2025). A Study on the Volume Changes of the Posterior Cervical Paravertebral Muscles After Unilateral Exposure Channel-Assisted Laminoplasty. Glob. Spine J..

[10] Chen S.W., Yeh K.T., Peng C.H., Chang C.M., Chen H.W., Yu T.C., Chen I.H., Wang J.H., Yang W.T., Wu W.T. (2024). Long-Term Outcomes of Modified Expansive Open-Door Laminoplasty Combined with Short-Level Anterior Cervical Fusion in Multilevel Cervical Spondylotic Myelopathy. Medicina.

[11] Wang B., Qu R., Liu Z., Zhao N., Pan S., Chen X., Zhao Y., Dang L., Zhou H., Wei F. (2025). Comparison of Postoperative Pain and Surgical Outcomes Between Three Types of Modified Muscle-Sparing Laminoplasty and Conventional Laminoplasty for Multilevel Degenerative Cervical Myelopathy. Glob. Spine J..

[12] Shen X., Xu C., Wang R., Zhang Z., Qi M., Zhang Y., Wu H., Liu Y., Chen H., Yuan W. (2023). Evaluation of Haplo-Paraspinal-Muscle-Preserving Technique to Prevent Postoperative Axial Pain in Cervical Laminoplasty. Int. J. Spine Surg..

[13] Wu W., Zhang S., Yan T. (2023). Initial clinical experiences of the muscle-preserving double door cervical laminoplasty with adjustable mini plates. Front. Surg..

[14] Yeh K.T., Lee R.P., Chen I.H., Yu T.C., Peng C.H., Liu K.L., Wang J.H., Wu W.T. (2016). The Midterm Surgical Outcome of Modified Expansive Open-Door Laminoplasty. BioMed Res. Int..

[15] Yeh K.T., Lee R.P., Chen I.H., Yu T.C., Peng C.H., Liu K.L., Wang J.H., Wu W.T. (2015). Laminoplasty with adjunct anterior short segment fusion for multilevel cervical myelopathy associated with local kyphosis. J. Chin. Med. Assoc..

[16] Arantes Júnior A.A., Silva Junior G.A., Malheiros J.A., Gonçalves F.F., Magaldi M., Santiago E., Nicolato A.A., Gusmão S.N. (2014). A new expansive two-open-doors laminoplasty for multilevel cervical spondylotic myelopathy: Technical report and follow-up results. Arq. Neuropsiquiatr..

[17] Yonenobu K., Abumi K., Nagata K., Taketomi E., Ueyama K. (2001). Interobserver and Intraobserver Reliability of the Japanese Orthopaedic Association Scoring System for Evaluation of Cervical Compression Myelopathy. Spine.

[18] Vernon H., Mior S. (1991). The Neck Disability Index: A Study of Reliability and Validity. J. Manip. Physiol. Ther..

[19] MacDowall A., Skeppholm M., Robinson Y., Olerud C. (2018). Validation of the Visual Analog Scale in the Cervical Spine. J. Neurosurg. Spine.

[20] Tang J.A., Scheer J.K., Smith J.S., Deviren V., Bess S., Hart R.A., Lafage V., Shaffrey C.I., Schwab F., Ames C.P. (2012). The Impact of Standing Regional Cervical Sagittal Alignment on Outcomes in Posterior Cervical Fusion Surgery. Neurosurgery.

[21] Kato S., Oshima Y., Matsubayashi Y., Taniguchi Y., Tanaka S., Takeshita K. (2019). Minimum Clinically Important Difference and Patient Acceptable Symptom State of Japanese Orthopaedic Association Score in Degenerative Cervical Myelopathy Patients. Spine.

[22] Li X., Yu H., Welle K., Gathen M., Zhang L., Xiao J., Kabir K. (2022). Comparative Effectiveness and Safety of Open-Door Laminoplasty, French-Door Laminoplasty, Laminectomy and Fusion, and Laminectomy Alone for Multilevel Degenerative Cervical Myelopathy: A Bayesian Network Analysis. Adv. Ther..

[23] Secer H.I., Harman F., Aytar M.H., Kahraman S. (2018). Open-door Laminoplasty with Preservation of Muscle Attachments of C2 and C7 for Cervical Spondylotic Myelopathy: Retrospective Study. Turk. Neurosurg..

[24] Jiang L., Chen W., Chen Q., Xu K., Wu Q., Li F. (2012). Clinical application of a new plate fixation system in open-door laminoplasty. Orthopedics.

[25] Shangguan Z., Chen G., Liu W., Li J. (2023). Clinical outcomes of modified versus traditional expansive open-door laminoplasty for cervical spondylotic myelopathy: A single-institution experience. J. Orthop. Surg..

[26] de Oliveira R.G., Fujii T., Daubs M.D., Su B.W., Louie P.K. (2026). Open Door Posterior Cervical Laminoplasty: A Step-by-Step Surgical Technique Guide by the Cervical Spine Research Society. Clin. Spine Surg..

[27] Yu Z., He D., Xiong J., Pan Z., Feng L., Xu J., Han Z., Gragnaniello C., Koga H., Phan K. (2019). Extensor muscle-preserving laminectomy in treating multilevel cervical spondylotic myelopathy compared with laminoplasty. Ann. Transl. Med..

[28] Zhang H., Zhu R., Yang H., Zhu X., Zhou F. (2012). Multifactor Analysis on the Outcomes of Cervical Spondylotic Myelopathy with Expansive Open-Door Laminoplasty. J. Int. Med. Res..

[29] Ivanov M. (2014). Modified open-door laminoplasty for the surgical treatment of cervical spondylotic myelopathy in elderly patients. Biomech. Concerns. Acta. Neurochir..

[30] Sakaura H., Ohnishi A., Yamagishi A., Ohwada T. (2019). Differences in postoperative changes of cervical sagittal alignment and balance after laminoplasty between cervical spondylotic myelopathy and cervical ossification of the posterior longitudinal ligament. Glob. Spine J..

[31] Schmeiser G., Bergmann J.I., Papavero L., Kothe R. (2022). Surgical Treatment of Multilevel Degenerative Cervical Myelopathy: Open-Door Laminoplasty and Fixation via Unilateral Approach. A Feasibility Study. J. Neurol. Surg. A Cent. Eur. Neurosurg..

[32] Nori S., Shiraishi T., Aoyama R. (2020). Comparison between muscle-preserving selective laminectomy and laminoplasty for multilevel cervical spondylotic myelopathy. Ann. Transl. Med..

