# Supplementary material for: Muscle-Preserving Versus Conventional Midline Expansive Open-Door Laminoplasty for Multilevel Cervical Spondylotic Myelopathy: A 10-Year Comparative Cohort Study
Source: Medicina (Kaunas). 2026 Oct 1;62(10):1902. doi: 10.3390/medicina62101902 (PMC13706684; doi:10.3390/medicina62101902)
Supplement: Supplementary file 1 [file medicina-62-01902-s001.zip › medicina-4474540-supplementary.pdf]

Table S1. Distribution of operative year by surgical group.

Operative year	Conventional, n (%)	Muscle-preserving, n (%)
2011	12 (28.6)	6 (10.3)
2012	9 (21.4)	11 (19.0)
2013	5 (11.9)	15 (25.9)
2014	10 (23.8)	16 (27.6)
2015	6 (14.3)	10 (17.2)
Total	42 (100.0)	58 (100.0)

Median operative year: 2012.5 (IQR, 2011–2014) in the conventional group and 2013 (IQR, 2012–2014) in the muscle-preserving group. Mann–Whitney U = 980.0, Z = −1.699, $p = 0.089$.

Table S2. Operative-year-adjusted longitudinal analyses of JOA, VAS, and NDI outcomes.

Part A. Global fixed effects.

Outcome	Effect	F	Numerator df	Denominator df	*p* value
JOA	Group × time	9.094	4	385.749	< 0.001
	Operative year	0.330	1	93.162	0.567
VAS	Group × time	5.559	4	384.457	< 0.001
	Operative year	11.453	1	91.949	0.001
NDI	Group × time	5.109	4	396.021	0.001
	Operative year	3.986	1	102.525	0.049

Part B. Operative-year coefficients.

Outcome	β per operative year	95% CI	*p* value
JOA	−0.0246	−0.110 to 0.060	0.567
VAS	0.134	0.056 to 0.213	0.001
NDI	0.266	0.002 to 0.531	0.049

Both techniques were represented in every calendar year from 2011 through 2015. MP, muscle-preserving.

Part C. Operative-year-adjusted estimated marginal means and between-group contrasts

JOA score

Follow-up	Conventional adjusted mean (95% CI)	MP adjusted mean (95% CI)	Adjusted difference, MP – Conventional (95% CI)	*p* value
3–6 months	13.708 (13.327 to 14.088)	13.925 (13.601 to 14.248)	0.217 (−0.284 to 0.718)	0.395
1 year	13.136 (12.756 to 13.517)	13.131 (12.808 to 13.455)	−0.005 (−0.505 to 0.496)	0.985
2 years	10.350 (9.970 to 10.731)	11.993 (11.670 to 12.317)	1.643 (1.142 to 2.144)	< 0.001
5 years	9.541 (9.160 to 9.921)	10.976 (10.653 to 11.300)	1.435 (0.935 to 1.936)	< 0.001
10 years	8.738 (8.347 to 9.128)	10.121 (9.786 to 10.456)	1.383 (0.867 to 1.899)	< 0.001

JOA estimated marginal means were obtained from a repeated-measures linear mixed model with a compound-symmetry covariance structure and restricted maximum-likelihood estimation. The model included surgical group, follow-up time, the group-by-time interaction, age, sex, baseline JOA score, number of decompressed levels, and operative year as fixed effects. Operative year was entered as a continuous variable coded as years since 2011. Between-group differences were calculated as muscle-preserving minus conventional. Time-specific pairwise comparisons were adjusted using the Sidak method. Denominator degrees of freedom were estimated using the Satterthwaite approximation.

VAS score

Follow-up	Conventional adjusted mean (95% CI)	MP adjusted mean (95% CI)	Adjusted difference, MP – Conventional (95% CI)	*p* value
3–6 months	4.056 (3.750–4.362)	3.098 (2.838–3.359)	−0.957 (−1.361 to −0.554)	< 0.001
1 year	4.770 (4.464–5.076)	3.426 (3.166–3.686)	−1.344 (−1.747 to −0.941)	< 0.001
2 years	5.222 (4.916–5.529)	3.529 (3.269–3.790)	−1.693 (−2.096 to −1.290)	< 0.001
5 years	5.603 (5.297–5.910)	3.995 (3.735–4.255)	−1.608 (−2.012 to −1.205)	< 0.001
10 years	5.946 (5.632–6.260)	3.718 (3.448–3.987)	−2.228 (−2.643 to −1.813)	< 0.001

NDI score

Follow-up	Conventional adjusted mean (95% CI)	MP adjusted mean (95% CI)	Adjusted difference, MP – Conventional (95% CI)	*p* value
3–6 months	13.727 (12.433 to 15.021)	12.795 (11.694 to 13.895)	−0.932 (−2.635 to 0.771)	0.283
1 year	14.489 (13.194 to 15.783)	12.777 (11.677 to 13.878)	−1.711 (−3.414 to −0.008)	0.049
2 years	17.036 (15.742 to 18.331)	11.795 (10.694 to 12.895)	−5.242 (−6.944 to −3.539)	< 0.001

5 years	18.346 (17.052 to 19.640)	14.209 (13.108 to 15.309)	−4.137 (−5.840 to −2.435)	< 0.001
10 years	20.592 (19.262 to 21.921)	15.395 (14.256 to 16.534)	−5.197 (−6.952 to −3.442)	< 0.001

Values are model-based estimated marginal means with 95% confidence intervals. Between-group differences were calculated as muscle-preserving minus conventional; therefore, positive differences favor the muscle-preserving group for JOA, whereas negative differences favor the muscle-preserving group for VAS and NDI. All models were adjusted for age, sex, the baseline value of the corresponding outcome, number of decompressed levels, and operative year. Operative year was entered as a continuous variable coded as years since 2011. The JOA model used a repeated-measures compound-symmetry covariance structure without an additional random intercept because the initially fitted random-intercept model produced a boundary estimate of zero for the random-intercept variance and a non-positive-definite Hessian. Time-specific pairwise comparisons were adjusted using the Sidak method. CI, confidence interval; JOA, Japanese Orthopaedic Association; MP, muscle-preserving; NDI, Neck Disability Index; VAS, visual analog scale.

Table S3. Hirabayashi JOA recovery rates by surgical group and postoperative assessment.

Follow-up	Conventional recovery rate mean ± SD	MP recovery rate mean ± SD
3–6 months	60.1% ± 17.3%	62.3% ± 15.8%
1 year	53.2% ± 17.2%	53.0% ± 16.9%
2 years	20.3% ± 15.8%	38.9% ± 16.7%
5 years	10.4% ± 15.8%	26.2% ± 16.6%
10 years	−0.7% ± 26.6%	15.8% ± 21.3%

The Hirabayashi recovery rate was calculated for each patient as [(postoperative JOA − baseline JOA)/(17 − baseline JOA)] × 100%. Negative values indicate deterioration below the preoperative JOA score. Values are presented as mean ± SD. At 10 years, the unadjusted mean difference between groups was 16.5 percentage points (95% CI, 6.3–26.7; $p = 0.002$).
